# Reaction Kinetics of the Synthesis of Polymethoxy Butyl Ether from n-Butanol and Trioxane with Acid Cation-Exchange Resin Catalyst

**DOI:** 10.3390/polym17233137

**Published:** 2025-11-25

**Authors:** Xue Wang, Linyu Lu, Qiuxin Ma, Hongyan Shang, Lanyi Sun

**Affiliations:** 1 College of Chemical Engineering, China University of Petroleum, Qingdao 266580, China; 2 School of Chemical Engineering, Shandong Institute of Petrochemical and Chemical Technology, Dongying 257061, China

**Keywords:** fuel additive, polymethoxy butyl ether, reaction kinetics, acid cation exchange resin, process optimization

## Abstract

Polymethoxy butyl ether (BTPOM*_n_*), a novel diesel additive developed for suppressing incomplete combustion emissions, was synthesized via an optimized batch slurry method employing n-butanol and trioxane (TOX) over NKC-9 acid cation-exchange resin (90–110 °C). A comprehensive kinetic model elucidated the reaction mechanism, addressing competitive pathways governing both main product formation and key side reactions—specifically polyoxymethylene hemiformals (HD*_n_*) and polyoxymethylene glycols (MG) generation. As the first detailed kinetic investigation of BTPOM*_n_* synthesis, this work provides a fundamental dataset and a robust predictive model that are crucial for process intensification and reactor design. Hybrid optimization integrating genetic algorithms with nonlinear least-squares regression achieved robust parameter estimation, with model predictions showing excellent agreement with experimental data. Thermal effects significantly influenced reaction rates, enhancing decomposition and propagation processes with increasing temperature. Optimal catalyst loading was identified at 3 and 6 wt.%, balancing reaction acceleration and byproduct suppression. Temperature-dependent equilibrium revealed chain length regulation through growth and depolymerization processes. This mechanistic understanding enables predictive reactor design for cleaner fuel additive synthesis. It provides critical insights for developing emission-control technologies in diesel engine systems.

## 1. Introduction

Diesel engines remain indispensable in industrial and transportation systems due to their superior thermal efficiency, high torque output, and fuel economy [1]. These characteristics make them particularly suitable for heavy-duty applications including freight transport, marine propulsion, and stationary power generation. Nevertheless, conventional diesel fuels derived from petroleum resources exhibit complex hydrocarbon compositions. Their combustion generates substantial nitrogen oxides and particulate matter emissions, abbreviated as NO*_x_* and PM respectively [2,3], which collectively endanger public health and ecological systems. NO*_x_* participates in photochemical smog formation and acid deposition, while PM induces respiratory diseases and degrades air quality. Mitigating these emissions represents a critical challenge for sustainable energy development. Furthermore, diesel engines operating at high altitudes face exacerbated combustion inefficiencies due to reduced atmospheric oxygen levels [4,5]. This oxygen-deficient environment promotes incomplete fuel oxidation, manifesting as ignition failures, accelerated component wear, and systemic performance deterioration.

Oxygenated fuels have emerged as viable diesel alternatives or blendstocks through their inherent oxygen content [6]. These compounds enhance combustion completeness by providing intramolecular oxygen, effectively suppressing carbon monoxide, unburned hydrocarbons, and soot formation. Among oxygenates, polyoxymethylene dimethyl ethers (PODE*_n_*) demonstrate exceptional potential due to their elevated cetane number, complete miscibility with hydrocarbons, high oxygen mass fraction, and favorable handling properties [7,8,9,10,11]. Numerous studies have validated the emission-reduction benefits of PODE*_n_*–diesel blends, particularly their capacity to simultaneously lower NO*_x_*, PM, and combustion noise outputs [12,13,14]. Lü et al. systematically demonstrated that optimized PODE*_n_* blends improve low-temperature operability by reducing cold filter plugging points while enhancing combustion efficiency and emission characteristics in modern diesel engines [15]. Lifecycle analyses further substantiate their environmental superiority: Rahman et al. reported well-to-wheel greenhouse gas intensities of 126.02 g CO_2_ eq/MJ for conventional diesel versus 89.34 g CO_2_ eq/MJ for biomass-derived PODE*_n_* [16], while Mahbub et al. quantified values as low as 18 g CO_2_ eq/MJ for forest residue pathways, representing an 85% reduction versus petroleum diesel [17]. Blending 10% PODE_1_ with diesel achieves notable emission reductions, decreasing greenhouse gases by 20–21% and smoke emissions by 30%.

Kinetic investigations of PODE*_n_* synthesis have employed diverse methodologies across reactor configurations. Zhang et al. investigated the reaction kinetics of PODE*_n_* synthesis from methanol and formaldehyde in a fixed-bed reactor [18,19]. They developed separate kinetic models for two catalysts with acidic cation-exchange resin, and metal oxide was developed to solve the equations via the fourth-order Runge–Kutta and Newton’s methods. Oestreich et al. explored PODE*_n_* synthesis kinetics with methanol and formaldehyde as reactants [20]. A model was created using Presto Kinetics simulated annealing algorithm and the hyperbolic method. It is applicable for formaldehyde–methanol ratios of 0.5–1.5 g/g and water content up to 23 wt.%. Liu et al. synthesized PODE*_n_* (*n* = 2–5) from methanol and paraformaldehyde [21]. A multi-step kinetic model was established with a power–function reaction model to determine the kinetics equation by measuring reaction rates at different temperatures. Results indicated each step had a reaction order of one, and activation energy decreased with increasing PODE*_n_* polymerization degree. Burger et al. studied the kinetics of PODE*_n_* synthesis via batch reaction of methylal and paraformaldehyde [22]. The reaction with pseudo-homogeneous and adsorption kinetic models was simulated to find that the latter better fitted experimental data and could model the batch PODE*_n_* synthesis well. Zhang et al. researched PODE*_n_* reaction rates in a continuous fixed-bed reactor [23]. A power–function rate equation for methylal and para-formaldehyde was developed based on consecutive reaction mechanism. It was solved using the fourth-order Runge–Kutta method and optimized parameters with particle swarm optimization. The equation effectively reflected reactant conversion and product distribution, offering a basis for reactor simulation and scale-up. Zheng et al. established a PODE*_n_* synthesis kinetics model in a batch reactor using paraformaldehyde and methylal [24]. The model exhibits good consistency with experimental data. Despite these advancements, PODE*_n_* implementation faces practical limitations. The pronounced density disparity between PODE*_n_* and conventional diesel causes phase separation at low temperatures and increases volumetric fuel consumption. These challenges underscore the critical need for developing novel polyether fuels with diesel-like density profiles and compatible molecular polarity, essential for seamless integration into existing engine systems.

Polymethoxy dibutyl ether (BTPOM*_n_*) demonstrates superior fuel properties compared to conventional PODE*_n_*, exhibiting a higher cetane number and elevated net calorific value, which directly translates to improved ignition performance and energy efficiency in compression-ignition engines [25]. BTPOM*_n_* presents enhanced compatibility as a diesel blending component with a density closely matching commercial diesel fuels. The molecular structure of BTPOM*_n_* comprises C_4_H_9_-O-(CH_2_O)*_n_*-C_4_H_9_ chains, where *n* represents the methoxy group polymerization degree. The synthesis of BTPOM*_n_*, often catalyzed by NKC-9 molecular sieve resin, was performed in a batch-stirred autoclave at moderate methylal/trioxane ratios. However, the reaction kinetics of BTPOM*_n_* have not been studied. It is not possible to perform direct comparisons with previous work due to different catalysts and reaction conditions used.

Therefore, the primary goal of this work is to establish the first comprehensive kinetic model for the acid-catalyzed synthesis of BTPOM*_n_* from n-butanol and trioxane. Experiments were conducted at various temperatures (90, 100, and 110 °C) and catalyst loadings (3 wt.% and 6 wt.%) to determine the reaction rate constants and activation energies. The novelty of this research lies in its detailed elucidation of the complex reaction network, including the main chain propagation pathways and key competing side reactions, which has not been previously addressed for the butyl-based polyethers. The developed model provides fundamental kinetic parameters and a predictive tool essential for reactor design and process intensification.

## 2. Materials and Methods

### 2.1. Materials

High-purity n-butanol (GC-grade, ≥98 mass%) and trioxane (GC-grade, ≥99 mass%) were procured from Shanghai Macklin Biochemical Technology Co., Ltd. (Shanghai, China). The macroporous cation-exchange resin catalyst NKC-9 was commercially sourced from Nanjing Guojin New Materials Co., Ltd. (Nanjing, China). Deionized water was generated in-house using a Millipore Milli-Q water purification system. Reference standards BTPOM_1_ (C_4_H_9_-O-CH_2_O-C_4_H_9_) and BTPOM_2_ (C_4_H_9_-O-(CH_2_O)_2_-C_4_H_9_) were provided by the Systems Engineering Institute at the Academy of Military Sciences (Beijing, China), serving as analytical standards for quantitative chromatographic analysis of BTPOM*_n_* oligomers.

### 2.2. Apparatus and Experimental Procedure

**Figure 1:**
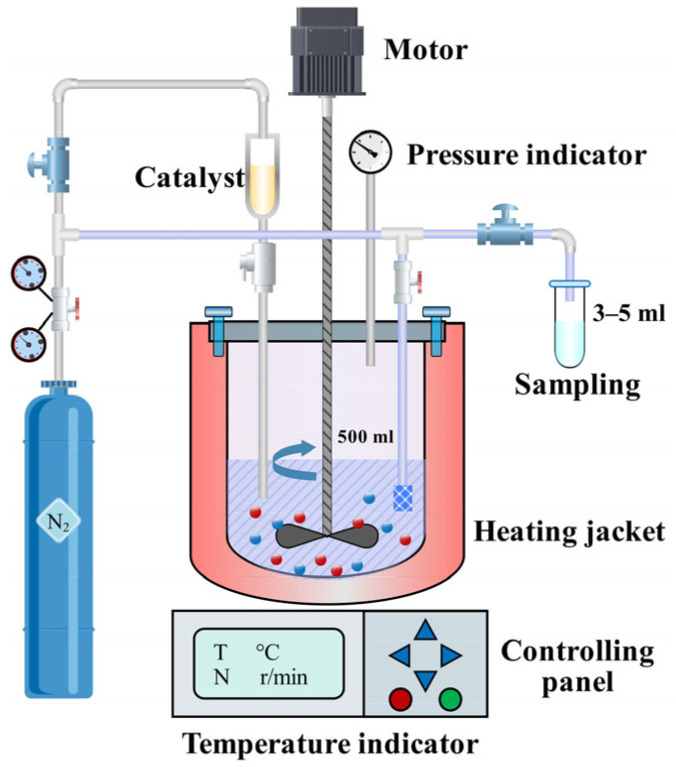
Schematic of the experimental setup for kinetic investigation.

Figure 1 presents the experimental setup for studying the reaction kinetics of poly(methoxy butyl ether) (BTPOM*_n_*) synthesis. The setup’s core is a 500 mL titanium alloy high-pressure autoclave (Model YZMR-4100D, Shanghai Yanzheng Experimental Instrument Co., Ltd., Shanghai, China) equipped with a dual-impeller mechanical stirrer featuring a 45° blade tilt angle and a 1:3 diameter ratio. This configuration generates intense turbulence to ensure thorough mixing of the NKC-9 resin catalyst with n-butanol and trioxane, reducing external diffusion limits and establishing intrinsic kinetic control. During premixing, the agitation rate was set to 300 r·min^−1^, increasing to 600 r·min^−1^ at reaction initiation. The reactant feed system utilized nitrogen pressurization initialized at 0.25 MPa via a digitally calibrated pressure regulator; following catalyst introduction through pressure differential injection, an adaptive pressure stabilization unit dynamically maintained reactor pressure between 1.0 and 1.1 MPa to suppress vaporization of low-boiling components. A dedicated sampling loop integrated with 5 μm sintered metal filters enabled intermittent extraction of catalyst-free liquid aliquots while retaining the solid-phase catalyst bed, and automated nitrogen backfilling immediately restored isobaric conditions following each sampling event. Temperature control leveraged a closed-loop feedback architecture combining Pt100 resistance temperature detectors with proportional–integral–derivative algorithms, achieving thermal uniformity within ±0.5 °C of setpoints, while parallel pressure management employed piezoelectric transducers interfaced with fast-response solenoid valves to constrain pressure fluctuations to ±0.02 MPa. The reactor assembly incorporated corrosion-resistant titanium alloy construction with multi-layered radial seals, augmented by continuous nitrogen inertization to eliminate oxidative catalyst deactivation through complete oxygen displacement.

Prior to the experiment, the 500 mL titanium alloy high-pressure autoclave was purged with nitrogen three times (each at 0.5 MPa for 5 min) to completely remove oxygen and prevent oxidative deactivation of the catalyst. Through the autoclave’s top feeding port, accurately weighed n-butanol (mass calculated per required molar ratio), trioxane, and deionized water were sequentially added, followed by premixing at 300 r·min^−1^ for 15 min to ensure uniform raw material dispersion. The NKC-9 resin catalyst pre-dried to constant weight (calculated based on 3 wt.% or 6 wt.% loading, accurate to 0.001 g) was injected into the autoclave via pressure difference, with nitrogen pressure maintained at 0.25 MPa during injection to avoid air ingress. After catalyst addition, the stirring rate was immediately increased to 600 r·min^−1^, and the heating system was activated to raise the temperature to the set value (90 °C, 100 °C, or 110 °C) at a heating rate of 5 °C/min. When the temperature reached the set value and stabilized (±0.5 °C), this was recorded as the reaction start time (t = 0), and the autoclave pressure was adjusted to 1.0–1.1 MPa via the pressure stabilization unit for stable maintenance. Sampling was performed at set time intervals: before sample collection, the first 5 mL of liquid in the sampling loop was discharged to rinse the pipeline and avoid cross-contamination, followed by collection of 3–5 mL of liquid aliquots for analysis. Given that the sampled volume was negligible relative to the total liquid volume in the reactor, no liquid was supplemented during the entire reaction process; after sampling, the nitrogen backfilling device was immediately activated to supplement nitrogen solely for restoring pressure to the set range. At the end of the reaction, heating was stopped, and the system was naturally cooled to room temperature; after depressurization, the reaction mixture was retrieved, and the catalyst was filtered, washed, dried, and weighed for subsequent stability analysis.

### 2.3. Analysis

Quantitative chromatographic analysis of liquid-phase components, including n-butanol, trioxane, BTPOM_1_, low-polymerization hemiformals (HD*_n_* with *n* ≤ 2), and BTPOM*_n_* oligomers (*n* ≤ 8), was performed using an Agilent 7820A gas chromatography system equipped with a flame ionization detector. The separation utilized an HP-1 capillary column (30 m × 0.32 mm ID, 0.25 μm stationary phase) under optimized temperature programming. Ethylene glycol diethyl ether served as the internal standard for peak area normalization, with its GC-grade purity exceeding 99.5% to ensure analytical accuracy [26].

Calibration factors for n-butanol, TOX, BTPOM_1_, and BTPOM_2_ were experimentally determined using certified reference materials. For higher oligomers (BTPOM*_n_* with polymerization degrees over 3), response factors were derived through linear extrapolation based on carbon chain length correlations. Water content quantification employed Karl Fischer coulometric titration, while formaldehyde concentration analysis followed the Chinese National Standard GB/T 9009-2011 potentiometric titration protocol [27]. Notably, the measured total formaldehyde concentration (W_OFA_) encompasses all chemically bound forms, including hydrate complexes and methanol adducts, with free monomeric formaldehyde constituting less than 2% of the total species. The equilibrium concentration of reactive formaldehyde (W_MFA_) was calculated using Equation (1), which accounts for temperature-dependent dissociation constants of formaldehyde derivatives.

(1) $$W_{MFA}=W_{OFA}-\frac{30}{88}W_{{HD}_{1}}-\frac{60}{112}W_{{HD}_{2}}$$

## 3. Reactions and Kinetic Model

### 3.1. Chemical Reactions

The catalytic synthesis of BTPOM*_n_* from n-butanol and trioxane over NKC-9 molecular sieve involves a complex multi-step reaction network. Trioxane serves as the formaldehyde precursor, undergoing depolymerization to generate reactive oxymethylene intermediates. This study systematically elucidates the reaction mechanism and kinetic behavior governing BTPOM*_n_* formation via formaldehyde oligomerization and subsequent etherification with n-butanol.

Chromatographic monitoring revealed distinct time-dependent progression in BTPOM*_n_* speciation following catalyst activation. Oligomer populations exhibited sequential emergence correlated with reaction time, while higher polymerization-degree homologues displayed monotonic concentration decay inversely proportional to chain length. Quantitative speciation analysis justified the exclusion of oligomers exceeding three repeating units from mechanistic modeling, as their cumulative molar fraction remained below 2% across experimental conditions. The kinetic model formalizes a consecutive chain propagation mechanism governed by the following elementary reactions [28,29]:

(2) $$TOX\overset{k_{1}}{\underset{k_{-1}}{⇄}}3FA$$

(3) $$2C_{4}H_{9}OH+FA\overset{k_{2}}{\underset{k_{-2}}{⇄}}BTPOM_{1}+H_{2}O$$

(4) $$BTPOM_{n}+FA\overset{k_{p,n}}{\underset{k_{d,n}}{⇄}}BTPOM_{n+1}$$

The inherent water content in the reaction system facilitated a competing catalytic pathway wherein formaldehyde underwent condensation to yield polyoxymethylene glycols (MG) as secondary products.

(5) $$H_{2}O+FA\overset{k_{MG}}{⇄}MG$$

*n*-Butanol underwent nucleophilic addition to formaldehyde, establishing the dominant pathway for polyoxymethylene hemiformal (HD*_n_*) generation. Analytical quantification identified HD_1_ and HD_2_ as the predominant intermediates, collectively representing over 85% of the total HD_n_ product distribution. The governing reaction sequence is expressed as follows:

(6) $$C_{4}H_{9}OH+FA\overset{k_{4}}{\underset{k_{-4}}{⇄}}HD_{1}$$

(7) $$HD_{1}+FA\overset{k_{5}}{\underset{k_{-5}}{⇄}}HD_{2}$$

### 3.2. Model Equations

The formulation of a molar concentration-based kinetic model was presented in detail. Component mass fractions (denoted as w*_i_*) were transformed into molar concentrations (C*_i_*) using Equation (8), assuming a constant reaction mixture density of 1.0 g/cm^3^. In this equation, M*_i_* is the molar mass of component *i*. Catalyst loading was defined as the mass ratio of catalyst (m_cat_) to total initial feedstock (m_feed_), expressed mathematically as w_cat_ = m_cat_/m_feed_.

(8) $$C_{i}=\frac{w_{i}}{M_{i}}$$

The kinetic model employed a pseudo-homogeneous phase approximation, presuming uniform dispersion of catalyst active sites in the liquid phase with unimpeded reactant accessibility. As for BTPOM*_n_* chain propagation kinetics, the forward and reverse rate constants denoted as k_3_ and k_−3_ were assumed independent of polymer chain length due to structural and mechanistic congruence across oligomerization steps. This assumption is mathematically represented as follows:

(9) $$\begin{array}{l} k_{p,1}=k_{p,2}=\ldots =k_{p,n}=k_{3}\\ k_{d,1}=k_{d,2}=\ldots =k_{d,n}=k_{-3} \end{array}$$

This strategic simplification effectively reduced the model’s parameter dimensionality while preserving essential reaction mechanisms. Consequently, the rate constants for the propagation steps of BTPOM*_n_* (*n* ≥ 1) are universally represented by k_3_ and k_−3_. This means that the coefficients mentioned in subsequent differential equations are numerically equivalent to k_3_ and k_−3_, reflecting the same fundamental kinetic parameters for chain elongation regardless of the specific oligomer involved. This approach enabled reliable parameter estimation through constrained optimization. Formaldehyde speciation analysis employed titration methods accounting for all chemically bound states. The total reactive formaldehyde concentration is denoted as W_MFA_, comprising both monomeric formaldehyde and polyoxymethylene glycol derivatives. Kinetic simplification assumed Reaction 5 as the sole equilibrium-controlled process governing MG formation, with K_MG_ representing its temperature-dependent equilibrium constant. Elementary reactions exhibit first-order dependence on all components except trioxane propagation steps. Trioxane chain propagation follows second-order kinetics. Based on the established reaction network and validated assumptions, the governing kinetic equations were formulated as follows:

(10) $$C_{FA}=\frac{C_{MFA}}{K_{MG}C_{H_{2}O}+1}$$

(11) $$\frac{dC_{C_{4}H_{9}OH}}{dt}=2W_{cat}k_{-2}C_{BTPOM_{1}}C_{H_{2}O}-2W_{cat}k_{2}C_{C_{4}H_{9}OH}C_{FA}-k_{4}C_{C_{4}H_{9}OH}C_{FA}+k_{-4}C_{HD_{1}}$$

(12) $$\frac{dC_{HD_{1}}}{dt}=k_{4}C_{C_{4}H_{9}OH}C_{FA}-k_{-4}C_{HD_{1}}-k_{5}C_{HD_{1}}C_{FA}+k_{-5}C_{HD_{2}}$$

(13) $$\frac{dC_{HD_{2}}}{dt}=k_{5}C_{HD_{1}}C_{FA}-k_{-5}C_{HD_{2}}$$

(14) $$\begin{array}{l} \frac{dC_{BTPOM_{1}}}{dt}=-W_{cat}k_{-2}C_{BTPOM_{1}}C_{H_{2}O}+W_{cat}k_{2}C_{C_{4}H_{9}OH}C_{FA}-W_{cat}k_{3}C_{BTPOM_{1}}C_{FA}\\ +W_{cat}k_{-3}C_{BTPOM_{2}} \end{array}$$

(15) $$\frac{dC_{TOX}}{dt}=-W_{cat}k_{1}C_{TOX}+W_{cat}k_{-1}C_{FA}^{2}$$

(16) $$\begin{array}{l} \frac{dC_{BTPOM_{2}}}{dt}=W_{cat}k_{3}C_{BTPOM_{1}}C_{FA}-W_{cat}k_{-3}C_{BTPOM_{2}}-W_{cat}k_{3}C_{BTPOM_{2}}C_{FA}\\ +W_{cat}k_{-3}C_{BTPOM_{3}} \end{array}$$

(17) $$\frac{dC_{BTPOM_{3}}}{dt}=W_{cat}k_{3}C_{BTPOM_{2}}C_{FA}-W_{cat}k_{-3}C_{BTPOM_{3}}$$

(18) $$\begin{array}{l} \frac{dC_{MFA}}{dt}=3W_{cat}k_{1}C_{TOX}-3W_{cat}k_{-1}C_{FA}^{2}-W_{cat}k_{3}C_{BTPOM_{1}}C_{FA}\\ +W_{cat}k_{-3}C_{BTPOM_{2}}-W_{cat}k_{3}C_{BTPOM_{2}}C_{FA}+W_{cat}k_{-3}C_{BTPOM_{3}}\\ -W_{cat}k_{3}C_{BTPOM_{3}}C_{FA}-W_{cat}k_{2}C_{C_{4}H_{9}OH}C_{FA}+W_{cat}k_{-2}C_{BTPOM_{1}}C_{H_{2}O}\\ -k_{4}C_{C_{4}H_{9}OH}C_{FA}+k_{-4}C_{HD_{1}}-k_{5}C_{HD_{1}}C_{FA}+k_{-5}C_{HD_{2}} \end{array}$$

(19) $$\frac{dC_{H_{2}O}}{dt}=-W_{cat}k_{-2}C_{BTPOM_{1}}C_{H_{2}O}+W_{cat}k_{2}C_{C_{4}H_{9}OH}C_{FA}$$

The variables C*_n_*, k*_n_*, and t correspond to the molar concentration of component *n* (mol/L), reaction rate constant for process *n*, and temporal progression (min), respectively. The temperature dependence of rate constants follows the Arrhenius equation, where A denotes the frequency factor, E_a_ the activation energy, and R the universal gas constant with the value of 8.314 J/(mol·K).

(20) $$k=Ae^{-\frac{E_{a}}{RT}}$$

Progressive liquid phase withdrawal during periodic sampling introduced cumulative mass depletion while maintaining constant catalyst mass. This resulted in a time-dependent escalation of catalyst loading relative to residual reactants, artificially accelerating reaction rates. The normalized temporal parameter t_N_ derived through Equation (21) was implemented to isolate intrinsic kinetic behavior from sampling artifacts. This correction accounts for the diminishing reactive phase volume by integrating the instantaneous liquid fraction remaining in the reactor:

(21) $$t_{N}=\frac{t_{R}m_{feed}}{m_{feed}-\sum_{i=1}^{N}m_{sample,i}}$$

where m_feed_ indicates the initial mass in grams of the reaction mixture containing BTPOM_1_, trioxane, and water, while m_sample,i_ corresponds to the cumulative mass in grams of liquid aliquots withdrawn at normalized reaction time t_R_ in hours. The nominal reaction time t_R_ was normalized to mitigate sampling-induced perturbations in catalytic kinetics. The methodology validated in prior studies of batch reaction systems.

### 3.3. Parameter Estimation

Reliable parameter estimation underpins the kinetic modeling framework. The parameter optimization procedure was implemented through numerical computing environment in MATLAB (Version R2022a, MathWorks, Natick, MA, USA). Governing differential equations were numerically integrated using the ode23s solver, selected for its stability in handling reaction networks with disparate timescales. The objective function minimized the sum of squared residuals between experimental and simulated molar concentrations across all monitored species. A hybrid genetic algorithm executed the global parameter search, systematically exploring the solution space to identify optimal kinetic constants while avoiding local minima convergence. Specifically, the genetic algorithm was implemented with a population size of 200 and a crossover fraction of 0.8, and was set to run for a maximum of 500 generations. This global search phase was designed to robustly identify a promising region within the parameter space, the results of which were then passed as initial guesses to a gradient-based nonlinear least-squares algorithm (lsqnonlin in MATLAB) for final refinement and rapid convergence.

(22) $$SSE=\sum_{i=1}^{N_{\mathrm{Data}}}{\left(C_{\exp}-C_{cal}\right)}^{2}$$

## 4. Results and Discussion

**Figure 2:**
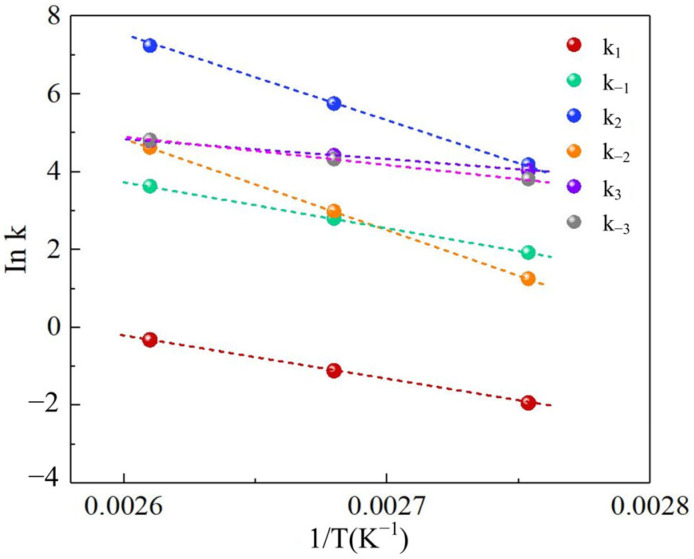
The relationships of rate constants of Reactions 2, 3 and 4.

**Figure 3:**
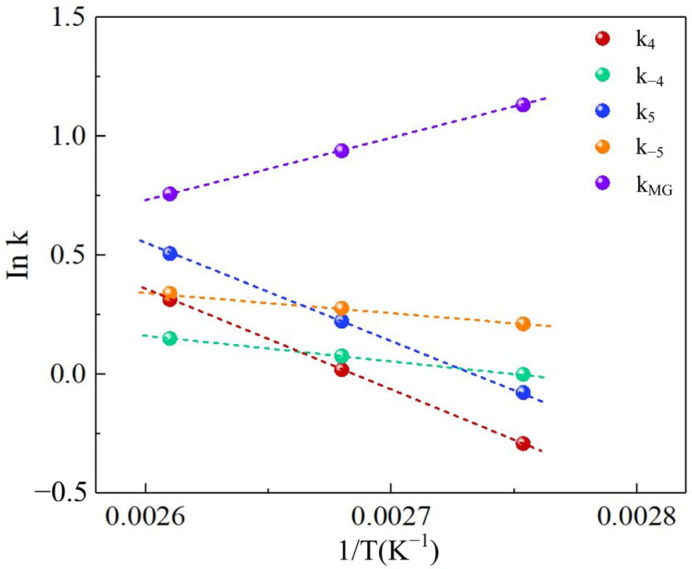
The relationships of rate constants of Reactions 5, 6 and 7.

Table 1 compiles the temperature-dependent rate constants (k*_i_*) and equilibrium constant (K_MG_) derived from parameter estimation. Figure 2 and Figure 3 demonstrate strong linear correlations between the natural logarithm of rate constants (ln k) and inverse temperature (1/T) for Reactions 1–3, validating adherence to Arrhenius behavior. The linear regression slopes yielded activation energies (E_a_) with regression coefficients exceeding 0.98. Similarly, equilibrium constant temperature dependence exhibits linear behavior consistent with thermodynamic expectations (R^2^ > 0.95).

Figure 2 quantitatively delineates the temperature-dependent kinetics and thermo-dynamics governing Reactions 2–4 through analysis of the Arrhenius equation. The forward rate constant k_1_ for trioxane depolymerization to formaldehyde in Reaction 2 increased exponentially from 0.14 to 0.72 L/(mol·min) over 90–110 °C (E_a_ = 93.69 kJ/mol), reflecting thermally enhanced monomer liberation. Simultaneously, the reverse rate k_−1_ surged from 6.86 to 37.78 L/(mol·min) (E_a_ = 98.67 kJ/mol). Thermodynamic analysis confirmed spontaneous formaldehyde generation despite accelerated recombination at elevated temperatures. The n-butanol-formaldehyde condensation in Reaction 3 exhibited pronounced thermal sensitivity, with k_2_ escalating 21-fold from 2.71 to 84.36 L/(mol·min) (E_a_ = 198.77 kJ/mol). It exhibits the high activation barrier, which can attribute to acid site-mediated transition state stabilization. The reverse reaction rate k_−2_ demonstrated analogous thermal acceleration from 6.20 to 175.12 L/(mol·min) (E_a_ = 193.25 kJ·mol^−1^), establishing a temperature-dependent equilibrium favoring BTPOM_1_ formation below 100 °C. In contrast, the chain propagation in Reaction 4 displayed moderated thermal responses, with k_3_ increasing from 1.73 to 4.64 L/(mol·min) (E_a_ = 57.18 kJ/mol) and k_−3_ rising from 21.94 to 59.71 L/(mol·min) (E_a_ = 57.91 kJ/mol). The low activation energy differential compared to Reactions 2–3 rationalizes the progressive polymer accumulation, driven by proton transfer mechanisms preferentially stabilizing chain elongation intermediates. These findings collectively establish a predictive framework for tailoring polymer molecular weight distributions through strategic temperature and catalyst modulation, underpinned by quantitative mapping of kinetic–thermodynamic tradeoffs across the reaction cascade.

Figure 3 delineates the temperature-governed kinetic–thermodynamic interplay between *n*-butanol-formaldehyde condensation and competitive water-formaldehyde polycondensation. The *n*-butanol-formaldehyde condensation generates hemiformal intermediates HD_1_/HD_2_ while water-formaldehyde polycondensation produces polyoxymethylene glycols. The forward rate constant k_4_ for the primary condensation step in Reaction 6 increased from 1.02 to 2.39 L/(mol·min) (E_a_ = 49.02 kJ/mol), demonstrating efficient acid-catalyzed hemiacetal formation via formaldehyde protonation-mediated nucleophilic addition. Conversely, the reverse reaction rate k_−4_ exhibited minimal thermal dependence from 0.17 to 0.50 L/(mol·min) (E_a_ = 62.71 kJ/mol), with thermodynamic analysis revealing a progressive equilibrium shift toward HD_1_ formation. Thermodynamic analysis of the competing water-formaldehyde pathway in Reaction 5 confirmed its exothermic nature (ΔH = −31.63 kJ/mol), with equilibrium constant K_MG_ decreasing from 70.92 to 41.05 L/(mol·min) as temperature increased from 90 to 110 °C, correlating with reduced MG yields at elevated temperatures. The secondary condensation step in Reaction 7 displayed attenuated kinetics, with k_5_ rising from 17.96 to 31.10 L/(mol·min) (E_a_ = 31.77 kJ/mol). This moderated acceleration stems from steric hindrance in HD_1_ intermediates and localized catalyst site saturation effects. The reverse reaction rate k_−5_ showed limited growth from 2.17 to 4.94 L/(mol·min) (E_a_ = 47.47 kJ/mol), further suppressing HD_2_ accumulation capacity.

Linear Arrhenius correlations (R^2^ > 0.98) for forward reactions contrasted with flattened profiles for reverse steps, indicating reverse kinetics governed by proton mobility limitations on catalyst surfaces. These insights establish an operational paradigm favoring moderate temperatures (90–100 °C) to minimize MG byproducts, providing critical thermal guidelines for process optimization.

**Figure 4:**
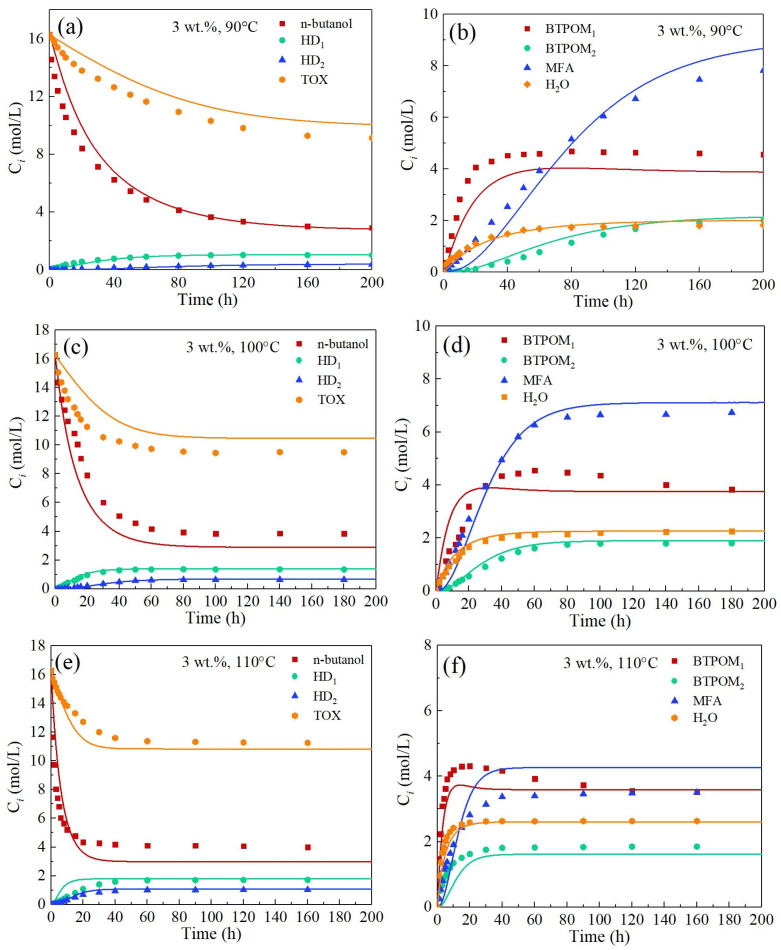
Comparison between experimental data (symbols) and model-calculated concentrations (solid lines) of all compounds at different reaction conditions and the parity plot with a catalyst loading of 3 wt.%: (**a**) *n*-butanol, HD_1_, HD_2_, TOX at 90 °C; (**b**) BTPOM_1_, BTPOM_2_, MFA, H_2_O at 90 °C; (**c**) *n*-butanol, HD_1_, HD_2_, TOX at 100 °C; (**d**) BTPOM_1_, BTPOM_2_, MFA, H_2_O at 100 °C; (**e**) *n*-butanol, HD_1_, HD_2_, TOX at 110 °C; (**f**) BTPOM_1_, BTPOM_2_, MFA, H_2_O at 110 °C.

Figure 4 elucidates temperature-dependent kinetic profiles and byproduct evolution in polymethoxy butyl ether synthesis (BTPOM_1_/BTPOM_2_) from n-butanol and trioxane at 3 wt.% catalyst loading. At 90 °C, slow trioxane decomposition (k_1_ ≈ 0.14 L/(mol·min)) required 200 h for complete conversion, with residual n-butanol stabilizing at 4 mol/L. Hemiformal intermediates HD_1_ and HD_2_ peaked at 1 and 0.5 mol/L, respectively, enabling gradual BTPOM_1_/BTPOM_2_ accumulation to 4/2 mol/L. Temperature elevation to 100 °C accelerated trioxane decomposition (k_1_ ≈ 0.33 L/(mol·min)), reducing n-butanol to 4 mol/L while amplifying HD_1_/HD_2_ maxima to 1.2/0.8 mol/L. Furthermore, BTPOM_1_/BTPOM_2_ concentrations increased to 4/2 mol/L, with formaldehyde peaking earlier for 7 mol/L at 80 h due to enhanced propagation kinetics. Rapid trioxane equilibration within 60 h (k_1_ ≈ 0.72 mol/min) drove the most n-butanol consumption under the condition of 110 °C. HD_1_/HD_2_ intermediates reached transient maxima of 2/1 mol/L. Target product concentrations peaked at 4/2 mol/L for BTPOM_1_/BTPOM_2_, with formaldehyde peaking for 3.5 mol/L at 40 h.

**Figure 5:**
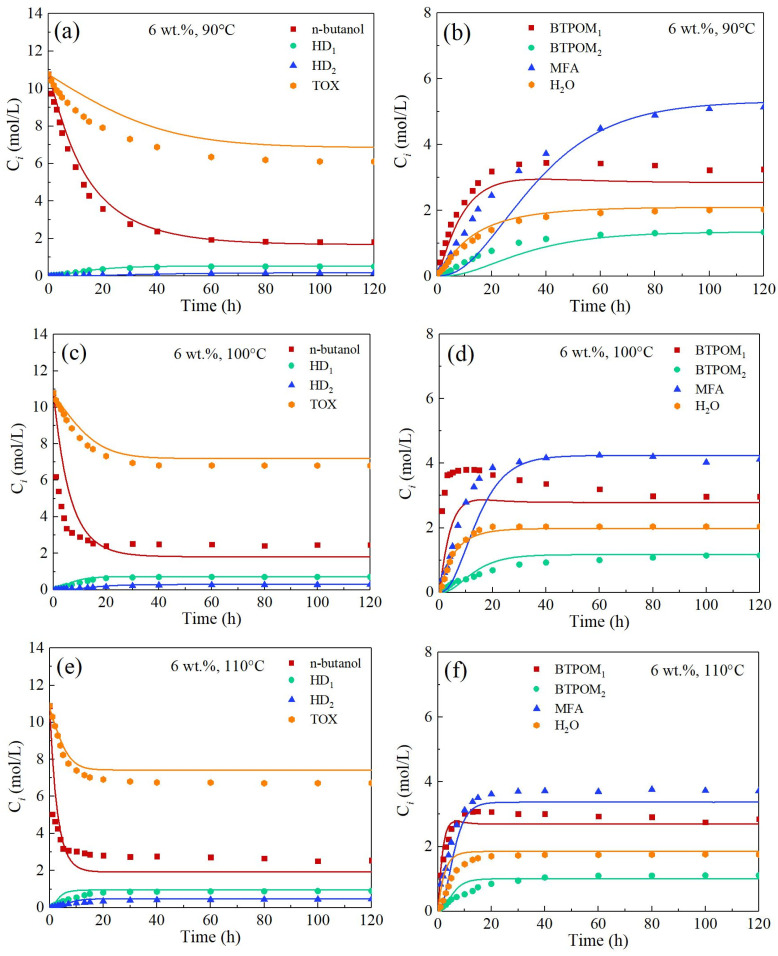
Comparison between experimental data (symbols) and model-calculated concentrations (solid lines) of all compounds at different reaction conditions and the parity plot with a catalyst loading of 6 wt.%: (**a**) *n*-butanol, HD_1_, HD_2_, TOX at 90 °C; (**b**) BTPOM_1_, BTPOM_2_, MFA, H_2_O at 90 °C; (**c**) *n*-butanol, HD_1_, HD_2_, TOX at 100 °C; (**d**) BTPOM_1_, BTPOM_2_, MFA, H_2_O at 100 °C; (**e**) *n*-butanol, HD_1_, HD_2_, TOX at 110 °C; (**f**) BTPOM_1_, BTPOM_2_, MFA, H_2_O at 110 °C.

Figure 5 illustrates the effect of 6 wt.% catalyst loading on the synthesis efficiency of polymethoxy butyl ethers (BTPOM_1_/BTPOM_2_) and byproduct dynamics across temperatures (90–110 °C). At 90 °C, trioxane decomposition kinetics resembled those under lower catalyst loadings, yet hemiformal byproduct concentrations (HD_1_/HD_2_) remained below 0.5 mol/L. Target product accumulation was limited by slow reaction kinetics, yielding only 3.5 and 1 mol/L of BTPOM_1_ and BTPOM_2_, respectively, after 120 h. At 100 °C, accelerated trioxane decomposition reached equilibrium within 30 h. BTPOM_1_/BTPOM_2_ concentrations increased to 3.6 and 1.2 mol/L, though the BTPOM_2_ fraction slightly decreased compared to the 3 wt.% catalyst system, suggesting a marginal yield reduction with higher catalyst loading.

Under 110 °C, trioxane achieved rapid equilibrium within 10 h. Peak BTPOM_1_/BTPOM_2_ concentrations reached 3 and 1 mol/L, with BTPOM_2_ constituting 25% of target products. Higher catalyst loading markedly accelerated BTPOM_1_ formation kinetics by facilitating proton transfer through increased active site density. This eliminated reaction induction periods and established rate-limited chain propagation as the dominant pathway. However, despite enhanced kinetics, overall target product yield slightly decreased. This phenomenon can be attributed to the two factors: (1) Competitive adsorption: High acid site density favors protonation of formaldehyde, which occupies active sites and diverts *n*-butanol toward side reactions. (2) Diffusion limitations: Aggregation of catalyst particles at 6 wt.% loading increases intraparticle diffusion resistance, hindering chain propagation within resin pores.

Comparative analysis of Figure 4 and Figure 5 reveals pronounced catalyst loading effects for 3 and 6 wt.% on reaction kinetics, product distribution, and model predictability from 90 to 110 °C. The 6 wt.% catalyst concentration accelerated TOX decomposition rates by 2.3-fold. This enhancement was attributed to improved proton transfer efficiency due to increased active site density. Accelerated BTPOM_1_ formation was particularly evident during the initial stages (t < 20 min). At 100 °C, the rate constant for BTPOM_1_ formation increased from 15.84 to 84.36 L/(mol·min). Elevated catalyst loading improved chain propagation kinetics at 110 °C. However, this intensification concurrently amplified competitive byproduct formation, resulting in a marginal yield reduction relative to the 3 wt.% system. However, this kinetic intensification concurrently amplified competitive byproduct formation pathways, culminating in a marginal yet consistent yield reduction relative to the 3 wt.% benchmark system. This nonlinear yield response originates from dual mechanistic constraints: (1) Competitive adsorption dynamics wherein elevated acid site density thermodynamically favors rapid protonation of formaldehyde, which saturates catalytic centers and sterically impedes n-butanol adsorption. This site blockade diverts n-butanol toward kinetically competitive side reactions, predominantly hemiformal condensations (HD_1_/HD_2_) and dehydration products. (2) Mass transfer limitations exacerbated by incipient catalyst particle aggregation at 6 wt.% loading, which induces mesopore occlusion and elevates intraparticle diffusion resistance. This architectural constraint imposes pronounced diffusional barriers on macromolecular intermediates, hindering chain propagation kinetics within the tortuous resin pore network and prematurely terminating BTPOM growth. Consequently, despite enhanced proton transfer efficiency, spatial congestion at molecular and particle scales imposes an inherent yield penalty on target product formation.

**Figure 6:**
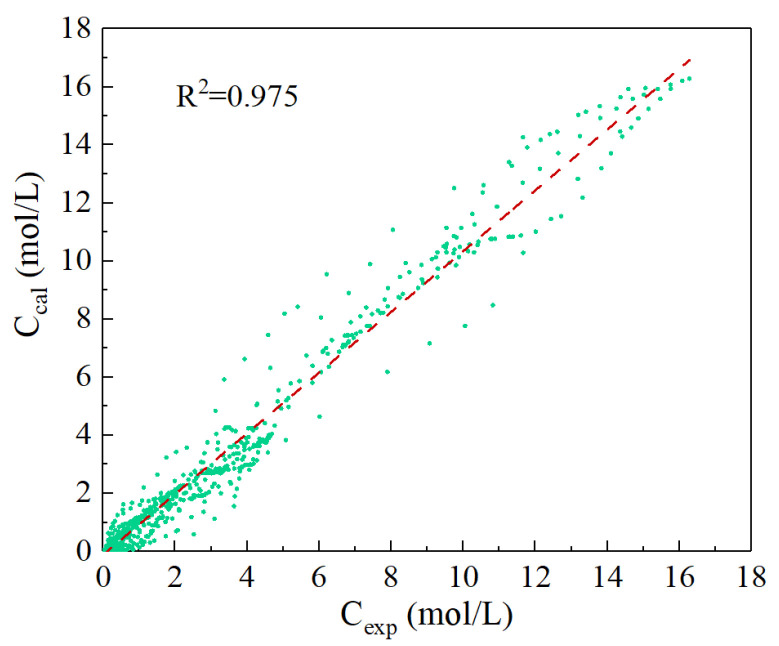
Parity plot comparing all experimental concentration data (C_exp_) with the corresponding model-simulated results (C_cal_).

The developed kinetic model, incorporating the optimized parameter values, enables comprehensive simulation of the polymethoxy dibutyl ether synthesis process. By numerically solving the system of differential equations with the determined rate constants under various experimental conditions, simulated concentration profiles were generated and rigorously validated against empirical data, as illustrated in Figure 6. The high consistency between model predictions and experimental measurements is quantitatively supported by a coefficient of determination (R^2^) of 0.975, calculated using Equation (23). The use of R^2^ as a goodness-of-fit metric is consistent with established practices in analogous kinetic modeling studies within the field. This strong agreement confirms the model’s capability to reliably describe the temporal evolution of both the main products and key byproducts, demonstrating its utility as a predictive tool for reactor design and process scale-up.

(23) $$R^{2}=1-\frac{\sum_{i=1}^{N}{\left(C_{i,\exp}-C_{i,cal}\right)}^{2}}{\sum_{i=1}^{N}C_{i,\exp}^{2}}$$

## 5. Conclusions

This study systematically elucidates the synergistic regulation of temperature and catalyst concentration in reaction pathways, product distribution, and selectivity in acid-catalyzed synthesis of polymethoxy butyl ethers from n-butanol and trioxane. Thermal elevation from 90 °C to 110 °C accelerated trioxane decomposition and condensation kinetics. The rate constant for trioxane decomposition (k_1_) exhibited a significant increase from 0.14 to 0.72 L/(mol·min) with rising temperature, while the condensation reaction, characterized by an activation energy of 198.77 kJ/mol, demonstrated a more than 20-fold enhancement in its rate constant (k_2_). It also suppressed polyoxymethylene glycol formation via thermodynamic limitations. Furthermore, the equilibrium constant for polyoxymethylene glycol formation (k_MG_) decreased from 70.92 to 41.05 L/(mol·min) over the temperature range studied, corresponding to a reaction enthalpy of −31.63 kJ/mol. This demonstrated dynamic equilibrium between kinetic activation and thermodynamic suppression. Catalyst loading increased from 3 to 6 wt.% enhanced reaction rates but marginally reduced target product yield. This work presents the first comprehensive kinetic model for BTPOM*_n_* synthesis, providing foundational data (rate constants, activation energies, equilibrium parameters) for the research community. The validated model serves as a powerful tool for simulating, scaling up, and optimizing the production process of this promising fuel additive. These findings establish scalable process design guidelines for clean fuel additive synthesis while advancing fundamental insights into acid-catalyzed etherification mechanisms, offering critical engineering implications for developing emission-reduction technologies in diesel engine systems.

## Figures and Tables

**Table 1:** Rate constants, activation energy values and pre-exponential factors for each reaction.

k*_i_*(K_MG_)	T/°C			Arrhenius Parameters	
	90	100	110	A	E_a_(ΔH)/kJ/mol
K_1_	0.14	0.33	0.72	4.30 × 10^12^	93.69
K_−1_	6.86	16.47	37.78	1.07 × 10^15^	98.67
K_2_	2.71	15.84	84.36	1.06 × 10^29^	198.77
K_−2_	6.20	34.46	175.12	3.89 × 10^28^	193.25
K_3_	1.73	2.87	4.64	2.90 × 10^8^	57.18
K_−3_	21.94	36.68	59.71	4.69 × 10^9^	57.91
K_4_	1.02	1.58	2.39	1.15 × 10^7^	49.02
K_−4_	0.17	0.29	0.50	1.79 × 10^8^	62.71
K_5_	17.96	23.81	31.10	6.67 × 10^5^	31.77
K_−5_	1.24	3.31	4.94	1.46 × 10^7^	47.47
K_MG_	70.92	53.56	41.05	0.002	−31.63

## Data Availability

The original contributions presented in this study are included in the article. Further inquiries can be directed to the corresponding author.

## Abbreviations

The following abbreviations are used in this manuscript:

BTPOM_n_	Polymethoxy butyl ether
HD_n_	Hemiformals
MGs	Polyoxymethylene glycols
PODE_n_	Polyoxymethylene dimethyl ethers

## References

[1] Tang Q.J., Ren B.Y., Wu J.P., Hu J.C., Fu J.Q., Zhang D.Q. (2025). Experimental study on diesel engine performance of tractor under transient conditions. Therm. Sci. Eng. Prog..

[2] Rana S., Saxena M.R., Maurya R.K. (2022). A review on morphology, nanostructure, chemical composition, and number concentration of diesel particulate emissions. Environ. Sci. Pollut. Res..

[3] Zhang W.B., Zhang Z.Y., Chen H., Ji Z.H., Ma Y.L., Sun F.Y. (2024). A review on performance, combustion and emission of diesel and alcohols in a dual fuel engine. J. Energy Inst..

[4] Liu J.L., Li Y.Y., Zhang C.H., Liu Z.T. (2022). The effect of high altitude environment on diesel engine performance: Comparison of engine operations in Hangzhou, Kunming and Lhasa cities. Chemosphere.

[5] Ceballos J.J., Melgar A., Tinaut F.V. (2021). Influence of environmental changes due to altitude on performance, fuel consumption and emissions of a naturally aspirated diesel engine. Energies.

[6] Rahiman M.K., Santhoshkumar S., Subramaniam D., Avinash A., Pugazhendhi A. (2022). Effects of oxygenated fuel pertaining to fuel analysis on diesel engine combustion and emission characteristics. Energy.

[7] Xue Z.Z., Zhu X., Zhang X.Y., Ma N., Abd-El-Aziz S.A. (2025). A comprehensive review on the production of polyoxymethylene dimethyl ethers as alternative synthetic fuel: From conventional indirect methodologies to sustainable direct routes. J. Environ. Chem. Eng..

[8] Liu J.H., Wang L.J., Wang P., Sun P., Liu H.F., Meng Z.W., Zhang L.D., Ma H.J. (2022). An overview of polyoxymethylene dimethyl ethers as alternative fuel for compression ignition engines. Fuel.

[9] Wang X.F., Li F., Gu X.H., Jin P. (2019). Research progress in polyoxymethylene dimethyl ethers as component of tailored diesel fuel. Mod. Chem. Ind..

[10] Chen H., He J., Hua H. (2017). Investigation on combustion and emission performance of a common rail diesel engine fueled with diesel/biodiesel/polyoxymethylene dimethyl ethers blends. Energy Fuels.

[11] Li B., Yoo K.H., Wang Z., Boehman A.L., Wang J. (2019). Experimental and numerical study on autoignition characteristics of the gasoline/diesel/ethanol and gasoline/diesel/PODE/ethanol fuels. Energy Fuels.

[12] Wang X.C., Chang X.L., Chen Z.M., Gao J.B., Zhao Y.W., Zou J., Xiao H.L. (2025). Spray and combustion characteristics of diesel/polyoxymethylene dimethyl ethers (PODE_n_) Mixtures: Role of CH_2_O chain length in PODE_n_. Fuel.

[13] Yang H., Zhang Y.Q., Li C., Yu F., Li X.H. (2021). Physicochemical characteristics of particulate matter emitted from the oxygenated fuel/diesel blend engine. Aerosol Air Qual. Res..

[14] Kowthaman C.N., Rahman S.M.A., Fattah I.M.R. (2023). Exploring the potential of lignocellulosic biomass-derived polyoxymethylene dimethyl ether as a sustainable fuel for internal combustion engines. Energies.

[15] Lv M., Zhao J.S., Ning Z. (2025). Evaporation and micro-explosion characteristics of PODE-diesel blended droplets. Trans. Chin. Soc. Agric. Eng..

[16] Rahman M.M., Canter C., Kumar A. (2015). Well-to-wheel life cycle assessment of transportation fuels derived from different North American conventional crudes. Appl. Energy.

[17] Mahbub N., Oyedun A.O., Kumar A., Oestreich D., Arnold U., Sauer J. (2017). A life cycle assessment of oxymethylene ether synthesis from biomass-derived syngas as a diesel additive. J. Clean. Prod..

[18] Zhang J.Q., Shi M.H., Fang D.Y., Liu D.H. (2014). Reaction kinetics of the production of polyoxymethylene dimethyl ethers from methanol and formaldehyde with acid cation exchange resin catalyst. React. Kinet. Mech. Catal..

[19] Zhang J.Q., Fang D.Y., Liu D.H. (2014). Evaluation of Zr-Alumina in production of polyoxymethylene dimethyl ethers from methanol and formaldehyde: Performance tests and kinetic investigations. Ind. Eng. Chem. Res..

[20] Oestreich D., Lautenschütz L., Arnold U., Sauer J. (2017). Reaction kinetics and equilibrium parameters for the production of oxymethylene dimethyl ethers (OME) from methanol and formaldehyde. Chem. Eng. Sci..

[21] Liu Y., Gao X.C., Gao H.X., Shi Z., Li P. (2014). Kinetics of Synthesis of Polyoxymethylene Dimethyl Ethers. Chem. React. Eng. Technol..

[22] Burger J., Ströfer E., Hasse H. (2012). Chemical equilibrium and reaction kinetics of the heterogeneously catalyzed formation of poly(oxymethylene) dimethyl ethers from methylal and trioxane. Ind. Eng. Chem. Res..

[23] Zhang J.Q., Tang B., Xia C.L., Liu X.B., Liu X.K., Fang D.Y., Liu D.H. (2015). Kinetics of Polyoxymethylene Dimethyl Ethers Synthesis from Methylal and Trioxymethylene. Chem. React. Eng. Technol..

[24] Zheng Y.Y., Tang Q., Wang T.F., Wang J.F. (2015). Kinetics of synthesis of polyoxymethylene dimethyl ethers from paraformaldehyde and dimethoxymethane catalyzed by ion-exchange resin. Chem. Eng. Sci..

[25] Song X.Z., Jiang J., An G.J., Wang Y.X., Cui S.N., Li B., Xie L.F. (2021). Design of new liquid component of fuel air explosive and its damage power. J. Propuls. Technol..

[26] Wang X., Fan N., Sun L.Y., Lin X.F., Xu Y.Q., Shang H.Y., Xia Y.F., An G.J. (2024). Synthesis and separation of polyoxymethylene dihexyl ether. J. China Univ. Pet..

[27] (2011). Formaldehyde solution for industrial use.

[28] Deng X.D., Cao Z.B., Li X.P., Han D.Y., Zhao R.X., Li Y.G. (2015). The synthesis of Polyoxymethylene dimethyl ethers for new diesel blending component. Synth. React. Inorg. Met.-Org. Chem..

[29] Qin W., Wang M., Hao Y., Li H.S., Zhao Y., Jiao Q.Z. (2014). Synthesis of polyoxymethylene dimethyl ethers catalyzed by Brønsted acid ionic liquids with alkanesulfonic acid groups. Ind. Eng. Chem. Res..

